# Printing strategies for scaling-up perovskite solar cells

**DOI:** 10.1093/nsr/nwab075

**Published:** 2021-04-28

**Authors:** Yulong Wang, Changyu Duan, Pin Lv, Zhiliang Ku, Jianfeng Lu, Fuzhi Huang, Yi-Bing Cheng

**Affiliations:** Xianhu Laboratory of the Advanced Energy Science and Technology Guangdong Laboratory, Foshan 528216, China; State Key Laboratory of Silicate Materials for Architectures, Wuhan University of Technology, Wuhan 430070, China; State Key Laboratory of Silicate Materials for Architectures, Wuhan University of Technology, Wuhan 430070, China; State Key Laboratory of Advanced Technology for Materials Synthesis and Processing, Wuhan University of Technology, Wuhan 430070, China; Xianhu Laboratory of the Advanced Energy Science and Technology Guangdong Laboratory, Foshan 528216, China; State Key Laboratory of Silicate Materials for Architectures, Wuhan University of Technology, Wuhan 430070, China; Xianhu Laboratory of the Advanced Energy Science and Technology Guangdong Laboratory, Foshan 528216, China; State Key Laboratory of Advanced Technology for Materials Synthesis and Processing, Wuhan University of Technology, Wuhan 430070, China; Xianhu Laboratory of the Advanced Energy Science and Technology Guangdong Laboratory, Foshan 528216, China; State Key Laboratory of Silicate Materials for Architectures, Wuhan University of Technology, Wuhan 430070, China; Xianhu Laboratory of the Advanced Energy Science and Technology Guangdong Laboratory, Foshan 528216, China; State Key Laboratory of Advanced Technology for Materials Synthesis and Processing, Wuhan University of Technology, Wuhan 430070, China; Xianhu Laboratory of the Advanced Energy Science and Technology Guangdong Laboratory, Foshan 528216, China; State Key Laboratory of Advanced Technology for Materials Synthesis and Processing, Wuhan University of Technology, Wuhan 430070, China; Department of Materials Science and Engineering, Monash University, Victoria 3800, Australia

**Keywords:** printing technology, perovskite solar cells, ink, large-area, crystallization, module stability

## Abstract

Photovoltaic technology offers a sustainable solution to the problem of soaring global energy demands. Recently, metal halide perovskite solar cells (PSCs) have attracted worldwide interest because of their high power conversion efficiency of 25.5% and great potential in becoming a disruptive technology in the photovoltaic industry. The transition from research to commercialization requires advancements of scalable deposition methods for both perovskite and charge transporting thin films. Herein, we share our view regarding the current challenges to fabrication of PSCs by printing techniques. We focus particularly on ink technologies, and summarize the strategies for printing uniform, pinhole-free perovskite films with good crystallinity. Moreover, the stability of perovskite solar modules is discussed and analyzed. We believe this review will be advantageous in the area of printable electronic devices.

## Teaser

This review details recent progress on perovskite solar cells fabricated by printing processes, and highlights how ink engineering realizes stable perovskite solar modules with high efficiency.

## INTRODUCTION

Solar energy is the most abundant energy on Earth and has the potential to play a major role in achieving carbon neutrality in the coming decades. Photovoltaic (PV) devices that can convert sunlight into electricity effectively and safely are the most widely deployed solar technology in the world. Currently, as a result of cumulative learning over many years, silicon solar cells dominate the global PV market. However, in recent years, metal halide perovskite-based solar cells (PSCs) have been identified as one of the most promising emerging photovoltaic technologies because of their high efficiency and possibility of high-volume production by printing processes [1].

The general perovskite chemical formula is ABX_3_, a typical corner-sharing BX_6_ octahedra forming an extended three-dimensional (3D) network, where the A site occupant is a monovalent organic or inorganic cation (such as methylammonium/CH_3_NH_3_^+^/MA^+^,  formamidinium/[HC(NH_2_)_2_]^+^/FA^+^ or cesium/Cs^+^), B is a divalent metallic cation (such as Pb^2+^ and Sn^2+^) and X is a halogen anion (I^−^, Br^−^ or Cl^−^) [2]. In 2009, MAPbX_3_ perovskites were first introduced as a sensitizer for dye-sensitized solar cells (DSSCs) by Miyasaka *et al.*, achieving a power conversion efficiency (PCE) of 3.8% [3]. However, the high solubility of metal halide perovskites in polar liquid electrolyte made the lifetime of these cells <1 hour. The poor structural stability of these materials results from weak electrostatic interaction between the A cation and the anionic B-X framework [4]. In 2012, this problem was overcome by Park *et al.* and Snaith *et al.* upon replacing the liquid electrolyte with a solid hole transporting material (HTM), 2,2^′^,7,7^′^-tetrakis[N,N-di(4-methoxyphenyl)amino]-9,9^′^-spirobifluorene (spiro-OMeTAD). Using this approach, PSCs with a lifetime >500 h and PCE around ∼10% were obtained, which had never been achieved through the use of solid-state DSSCs [5,6]. Thereafter, the feverish research activity on hybrid perovskite is among the hottest topics in chemistry and materials science. PSCs have been separated from DSSCs and become a new category of emerging PV technology in the National Renewable Energy Laboratory (NREL)’s Best Solar Cells Efficiencies Chart in 2014, in which École Polytechnique Fédérale de Lausanne marked the first record of 14.1% for PSCs with a sequential deposited perovskite film [7]. After that, efficiency values were increased through strategies such as perovskite composition engineering and thin film deposition process optimization [2]. In 2018, You *et al.* from Institute of Semiconductors, Chinese Academy of Sciences successfully fabricated PSCs with efficiencies >23% by replacing the electron transporting layers (ETLs) from mesoporous TiO_2_ to planar SnO_2_, allowing for the possibility of making high-efficiency PSCs at low temperature (<200°C) [8]. As of August 2020, a state-of-the-art PSC demonstrated a PCE of 25.5% by Ulsan National Institute of Science and Technology, and it does not look likely that there will be any slow-down in research into this hot topic [9].

**Figure 1.**
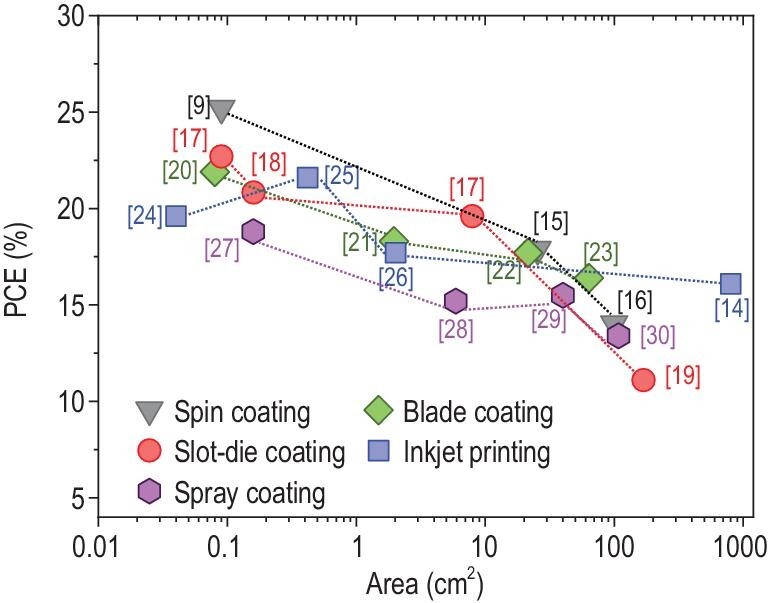
The champion PCEs determined by *J−V* measurements with respect to the active area for different fabrication processes [9,14,15–30]. For the modules, aperture areas are used to give a consistent overview.

To bring the PSC technology to a market which has been dominated by silica solar cells for decades, there are many challenges yet to be overcome, such as large-area module fabrication and operational stability-two factors that often display an inverse inter-dependency [10]. Recently, great advancements have been made regarding the stability issues of PSCs. A group led by Han *et al.* from Huazhong University of Science and Technology, China reported that their fully printed PSCs with triple-layer scaffold of TiO_2_/ZrO_2_/carbon passed the international aging standard of IEC61215:2016, which is for mature PV technologies [11]. In this regard, there have been endeavors to develop scalable coating/printing techniques, such as blade coating, slot-die coating, inkjet printing (IJP) and spray coating for high-efficiency perovskite solar modules. In March 2019, NREL released the Champion Photovoltaic Module Efficiency Chart, which is specifically for the development of solar modules. In 2018, WonderSolar launched a 110 m^2^ perovskite PV system with screen-printed triple mesoscopic PSC modules, which has good stability but low efficiency (∼10%) [12]. Another Chinese company, Utmolight Corp., achieved a PCE of 20.5% for a mini-module with an active area of 63.98 cm^2^, which led the world record efficiency of the perovskite mini-module [13]. In 2020, Panasonic Corp. (Japan) announced the world's highest PCE of 17.9% for a larger perovskite module (>800 cm^2^) [14]. Although great progress has been made, the PCE of perovskite modules still lags behind the state-of-the-art small area (∼0.1 cm^2^) cells fabricated by spin coating (see Fig. 1) [9,15–30]. Consensus has been reached that the quality of the large-area films, particularly the perovskite, are generally poorer than the smaller ones, which not only decreases the device efficiency, but also hinders the disclosure of failure mechanism of modules [31].

Recently, many reviews have been published on the topic of perovskite film deposition techniques/mechanisms, such as solvent engineering and additives-engineering [32–35], whereas discussions about ink engineering for printing high-quality perovskite film as well as other function layers are rare. In this review, we first provide a background to the printing/coating methods that have been developed for scaling-up high-efficiency perovskite modules, primarily over the past 5 years. Then, the effect of the ink recipe on the film quality, the underlying physical and chemical mechanisms, as well as the resulting perovskite module performance are discussed. Moreover, we present the technical feasibility of printing other layers besides perovskite layers, including hole transporting layers (HTLs) and electron transporting layers, which could enable rapid and mass production of PSCs. Finally, we discuss recent progress on roll-to-roll (R2R) printing and the stability issues of perovskite modules, and detail the prospects for mass production of perovskite solar modules in the near future.

## PRINTING TECHNIQUES

**Figure 2.**
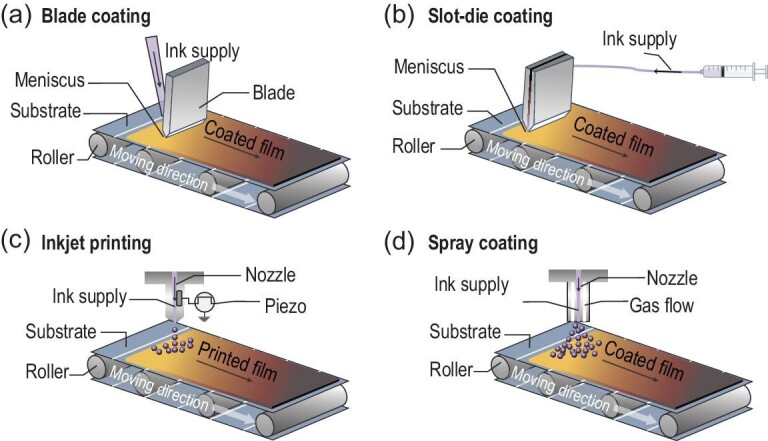
Scalable methods for PSCs deposition: (a) blade coating; (b) slot-die coating; (c) inkjet printing; (d) spray coating.

The quality of perovskite and charge transporting films is commonly directed by the film-fabrication process [36]. For example, when a MAPbI_3_ film was deposited onto a compact TiO_2_ (ETL)-coated substrate using a conventional deposition process, it formed a dendritic microstructure with voids and pin-holes, resulting in poor performance for solar cells [37]. However, when gas-blow or anti-solvent was introduced during the deposition process, compact and uniform perovskite films could be achieved [38,39]. Therefore, various techniques, such as blade coating [23], D-bar coating [40], gravure-printing [41], slot-die coating [42], IJP [43] as well as spray coating [28] (see Fig. 2) with different ink recipes and treatments have been adapted for printing/coating large area perovskite and functional layers.

### Blade coating and D-bar coating

Blade coating and D-bar coating are close analogues, which have attracted great research interest in printing thin films [44]. For blade coating (Fig. 2a), the ink is directly loaded onto the substrate and a knife-type coater is used to spread ink on the substrate. Therefore, the thickness of the film mainly depends on the meniscus forming between blade and substrate, as well as the ink concentration. The meniscus can be tailored by the geometry of blades, the gap between the blade and the substrate, the moving speed of the blade relative to the substrate, the viscosity of inks, the substrate wettability, *etc*. To date, PSCs with blade-coated perovskite have demonstrated PCEs >21% for small-area and 18.6% for modules (30–60 cm^2^) [14]. Moreover, functional layers, such as HTLs, *i.e.* spiro-OMeTAD [45], poly(triarylamine) (PTAA) [44], poly(3,4-ethylenedioxy thiophene):polystyrene sulfonate (PEDOT:PSS) [46], and ETLs, *i.e.* [6,6]-phenyl-C61-butyricacid methyl ester (PCBM) [46] and TiO_2_ [45] have been successfully blade-coated. All-blade-coated small-area PSCs have been demonstrated to be as efficient as 19.6%, showing great potential for future mass-production application [47].

Fast and large-area blade coating is important to make PSCs economically competitive. Generally, it is a prerequisite to achieve compact and uniform perovskite films with a thickness >400 nm for PSCs with high current density. However, when wet film is drying over a large-area substrate, microscale fluid flows towards perovskite nuclei and the surface tension increases, forming ‘islands’ about 10–200 *μ*m size. Deng *et al.* demonstrated that surfactants and preheating of the substrate can effectively suppress the island growth by reducing the surface tension, thus overcoming the Landau-Levich issue [44]. The molecular structure of surfactants could strongly affect the fluid and particle flow during the drying of the wet film, therefore the homogeneity of coated films. For example, among amphoteric L-*α*-phosphatidylcholine, non-ionic polyethylene glycol sorbitan monostearate (Tween 60), anionic sodium dodecyl sulfate and cationic didodecyldimethylammonium bromide, only the ink containing ∼20 ppm of L-*α*-phosphatidylcholine produced shiny, flat and smooth perovskite films [44].

### Slot-die coating

Slot-die coating is a technique classed as pre-metered coating. It is well suited to scaling-up perovskite as well as other layers in the device stack [42]. A slot-die-coater is generally composed of a slot-die head with an upstream and a downstream die (see Fig. 2b). Upon filling ink into the slot-die head through a feed slot, a liquid bridge between the coating head and the substrate is formed. While the coating head moves along the substrate, a uniform wet thin film is obtained. With a given coating width, the thickness of the wet film can be controlled by the flow of ink to the coating head, the speed at which the substrate moves past the head, as well as the concentration of the ink. Having a good wet film, optimization of the nucleation and crystal growth process is critical to obtain dry films with good coverage and uniformity [48].

Pioneering works on slot-die coating have proposed many models, such as the capillary model, viscous model and visco-capillary model to illustrate the operating processes in this printing technology. Watson *et al.* [42] summarized several common challenges for slot-die coating: (1) ‘low-flow’ limit caused discontinuity in the wet film, for example, breaks of the liquid bridge between coating head and substrate; (2) ‘over flow’ flooding, where the flow of ink to the head is more than the coating speed, resulting in mismatch of pre-metering and expected film thickness; (3) air-entrainment defects, associated with the breakup of the upstream meniscus leading to ‘bubbles’ within the wet film and areas of uncoated substrate. Therefore, a major concern in slot-die coating processes is how to determine the operating limits to set an appropriate range of operating parameters, including coating speed, flow rate, coating gap, liquid viscosity, surface tension, *etc.* [49].

In 2015, slot-die coating was first applied to fabricate PSCs with a device configuration of ITO/ZnO/MAPbI_3_/P3HT/Au [50]. The MAPbI_3_ film was obtained by sequentially slot-die coating a PbI_2_ film and then a MAI layer on top. By adjusting the processing-temperature of MAI coating, PSCs with efficiency of 12.0% were achieved [50]. Moreover, both charge transporting layers, including the ETL (ZnO) and HTL (P3HT) were coated by slot-die. Pre-heating the substrate and gas-blowing are generally the two methods used to dry the wet films, which significantly influence the film morphology and device performance. For example, along pre-heated substrate, gas-blowing and NH_4_Cl additive, Zuo *et al.* achieved high-quality perovskite films on both glass and flexible PET substrates at ambient. Ultimately, with a device configuration of ITO/PEDOT:PSS/MAPbI_3_/PCBM/Ag, champion PCEs of 15.6% for glass PSCs and 11.2% for flexible PSCs have been achieved [51].

Challenges in commercialization of PSCs include developing scalable deposition strategies for all layers over large-area substrates, not only the perovskite photoactive layer, but also ETLs, HTLs and electrodes. A complete printing/coating process for the whole device fabrication processes would be beneficial for high-throughput, mass production of PSCs. One merit of slot-die coating is that not only the substrate but also the slot head can be heated to an elevated temperature to tailor the viscosity and the solubility of the solids in inks [34,52]. For example, Song *et al.* deposited the PPDT2FBT:PC_71_BM organic film using a controlled temperature matrix of solution (slot head) and substrate (stage) temperatures in a range of 25°C to 100°C. They found that an optimal temperature condition (80°C head and 80°C substrate) for the slot-die coated organic solar cells (OSCs) showed an impressive PCE of 7.6% without using any additives or other processing treatments, implying a promising application for organic charge transporting layer printing in PSCs [53]. By adjusting the fluid dynamic, Bu *et al.* slot-die-coated an efficient ETL layer with a commercially available aqueous SnO_2_ colloidal solution diluted in water and isopropanol, achieving 15.2% efficiency of a flexible solar module (5 cm × 6 cm) [54]. Inorganic HTL layer, NiO_x_ film was successfully slot-die coated by spreading a precursor ink containing nickel acetate tetrahydrate, ethanolamine, tetramethylammonium hydroxide pentahydrate and ethanol [55]. The fully slot-die coated PSCs with an area of 12 cm × 12 cm showed efficiency of 11.0%. Another advantage of slot-die coating over blade coating is that the roughness of the substrate has negligible impact on the coating process. Therefore, high-quality charge transporting layers such as PTAA, as thin as a few nanometers can be slot-die-coated. Recently, Subbiah *et al.* successfully slot-die-coated a MAPbI_3_ perovskite ink onto the textured substrate, which resulted of a 2-terminal perovskite/silicon tandems cell with PCE of 23.8% [56]. Two strategies have been used to overcome the micrometer roughness of the texture substrate. First, the conventional DMF solvent was replaced by low-boiling-point acetonitrile with methylamine mixture (MA in methanol), which ensured swift evaporation of the solvent. Second, L-*α*-phosphatidylcholine as a surfactant was added to the perovskite ink to improve the wettability and ink–substrate interaction on substrates. Such results demonstrate the promise of slot-die coating for scaling-up PSCs in both single and multi-junction implementations.

### Inkjet printing

Inkjet printing (IJP) is familiar to most people given its commercial success in office printers. Among various inkjet systems, piezo-driven drop-on-demand (DoD) IJP is reported to be a reliable, versatile and cost-effective industrial production technology that is suitable for printed electronic and optoelectronic materials, such as metal nanoparticles, polymers and perovskite [57]. It also allows for printing thin films with fine patterns at high resolution under ambient conditions [57,58]. The general principle of a DoD inkjet printer is shown in Fig. 2c, where the printer head is located above the substrate table, with at least one of them being movable. The printer head is connected to a continuous supply of ink. By applying a short voltage pulse to the piezoelectric element of the print head, a single spherical droplet is created as required. Mathies *et al.* suggested that a systematic IJP printing development process includes an analysis of the ink wetting behavior on the individual substrates as well as the adhesion of the dried layer [43]. Therefore, a ‘Magic Triangle’ including ink, substrate and print head was proposed to precisely search IJP protocols for efficient PSCs. In 2014, Yang *et al.* firstly used a two-step IJP process to fabricate PSCs, in which carbon black and MAI mixed ink were printed on a spin-coated PbI_2_ layer. The IJP approach demonstrated better crystallinity and an improved MAPbI_3_/carbon interface in comparison with the spin-coated ones, which ultimately led to a higher PCE of 11.6% [59]. Recent news from Panasonic Corp. about the world's highest PCE of 17.9% for large perovskite solar module (>800 cm^2^) was likely facilitated by an IJP method [9]. Such a result demonstrates that IJP has significant promise in perovskite module application.

Apart from printing parameters, solvents determine the precursor solubility of perovskite components, the type of lead-complexes and also the drying behavior through their evaporation [24]. Therefore, solvents used with regard to concentration, viscosity, surface tension, flow dynamics, *etc.*, significantly influence the IJP process window. Generally, solvents of strong Lewis base with great coordination capability are good candidates for IJP inks to achieve high-quality perovskite films [60,61]. Eggers *et al.* reported that homogeneous and pinhole-free thin films could be printed with high coordinating solvents such as DMSO, while pure GBL or DMF caused formation of island-shaped polycrystalline perovskite domains or isotropic growth of dendritic and nanowire structures [25]. A mixture solvent of GBL/DMF/DMSO enabled achievement of a high efficiency of 21.6% for IJP-based PSCs. Li *et al.* synthesized a PbX_2_-DMSO (X = Br, I) complex as a precursor and used mixed solvent NMP/DMSO to control the crystallization during the IJP process. As a result, a homogeneous Cs_0.05_MA_0.14_FA_0.81_PbI_2.55_Br_0.45_ perovskite film with large grain sizes was obtained, which delivered a PSC with high PCE of 19.6% [24].

A full IJP process is beneficial for high-throughput and mass-production of PSCs. For this purpose, IJP of HTL and ETL inks are required. Schackmar *et al.* developed an IJP process for PSC in *p-i-n* architecture with all-IJP absorber and charge transport layers [62]. A uniform NiO_x_ HTL was obtained by firstly printing a nickel (II) acetate dihydrate (NiAc) wet film and subsequently annealing it in ambient. Then, a triple-cation Cs_0.1_MA_0.15_FA_0.75_PbI_2.55_Br_0.45_ perovskite layer with micrometer thick columnar crystal structures was printed on the NiO layer. A thin layer of PCBM was also inkjet-printed with the ink based on 1,2-dichlorobenzene:mesitylene (*o*DCB:MT) mixed solvent, in which the aromatic solvents allow IJP processing on the underlying multicrystalline perovskite layer and MT is used for reducing the high surface tension of *o*DCB. Finally, an interfacial layer of 30 nm BCP was printed with resolutions of 1000 dpi. This PSC showed PCE of 17.2% with a stable continuous-operation (>40 h) stability at 85°C, which is one of the most stable PSCs by the IJP method.

Generally, most of the researchers focused on one-step IJP processing in PSC fabrication, in which the ink contains all perovskite compositions to crystallize into perovskite structure. A fast and controllable removal of solvents and volatile additives by thermal annealing seems to be necessary to achieve high-quality perovskite films for PV devices. Therefore, development of slow-drying inks that allow a delayed processing time to separate printing wet-film and post treatment, such as thermal annealing and vacuum drying appear to be an important approach for further progress. One more challenge with IJP is the adhesion between the perovskite solution and substrate, which depends on surface roughness of the substrates and electrostatic/chemical interactions at the interface. For example, most of the polymer substrates for flexible PSCs have long molecular chains that contain very few bonding points for adhesion. Surface modification/treatments such as plasma treatment and atomic/molecular layer deposition may be needed to modify the adhesion between flexible substrates and inks for charge transporting layers.

### Spray coating

Spray technology is a solution-based deposition technique that has been used in many applications, such as fabricating thin films and automotive painting. The main merits of spray coating are the compatibility with non-planar surfaces, controllable deposition and high film uniformity [63]. Typically, an ultrasonic nozzle vibrating at tens of kHz is used to create a mist of ink droplets, which are directed as a spray onto the substrate using a compressed gas-jet (Fig. 2d). Then, these droplets adhere on the substrate to create a continuous wet-film that dries as a uniform film. The size and uniformity of the droplet are of crucial importance to achieve acceptable uniformity of the film and are affected by the properties of the coating solution (including the viscosity and surface tension), the nozzle type, the flow rate through the nozzle and the air (or gas) pressure. Therefore, introducing a solvent with low surface tension into the ink is an effective way to reduce the contact angle because of the Marangoni effect, while pre-heating the substrate is another strategy to decrease the surface tension and contact angle of the ink.

For non-contact droplet-based methods such as IJP and spray coating, inks reach the substrate in the form of separate droplets. The physical properties of the ink, such as viscosity, density, surface tension, contact angle, *etc.*, have to be within distinct processing windows [43]. Several dimensionless numbers consolidating these properties are frequently used to quantitatively characterize the printability of inks, such as Reynolds (*Re*, Eq. 1), Weber (*We*, Eq. 2) and Ohnesorge (*Oh*, Eq. 3) dimensionless parameters. The definitions of *We*, *Re*, *Oh* and inverse Ohnesorge number *Z* are expressed as follows:

(1) \begin{equation*}Re\ = \frac{{\textit inertial\ {\textit forces}}}{{\textit viscous\ {\textit forces}}}\ = \frac{{\rho * v *l}}{\mu },\ \end{equation*}

(2) \begin{equation*}We\ = \frac{{\textit inertial\ forces}}{{\textit viscous\ forces}}\ = \frac{{\rho * {v^2} * l}}{\gamma },\ \end{equation*}

(3) \begin{eqnarray*}Oh &=& \frac{{\textit viscous\ forces}}{{\sqrt {\textit inertial\ forces\ *\ surface\ forces} }}\nonumber\\ & =& \frac{{\sqrt {We} }}{{Re}}\ = \frac{\mu }{{\sqrt {\rho *\gamma *l} }},\ \end{eqnarray*}

(4) \begin{equation*}Z\ = \frac{1}{{Oh}}{\rm{\ }} = \frac{{Re}}{{\sqrt {We} }}\ = \frac{{\sqrt {\rho *\gamma *l} }}{\mu },\ \end{equation*}

where *ρ* refers to the ink density, *v* to the drop velocity, *l* to a characteristic nozzle diameter, *μ* to the dynamic viscosity and *γ* to the surface tension of the ink. According to Yang and Fan, a value of *Z* in the range of 1–10 is required to form stable and separated droplets [57]. With the rheology properties limitation, the optimal ink viscosity and surface tension are proposed to be in the range of 1–15 mPa · s and 20–70 mN/m, respectively. Ahmadian-Yazdi *et al.* found that surface tensions of perovskite inks are comparable to their solvents, whereas the viscosities of the inks are generally larger than the latter [64].

Barrows *et al.* first introduced spray coating for fabrication of perovskite thin films in 2014, reporting a champion PCE of 11.1% for the small-area PSC (aperture area 0.025 cm^2^). They found that substrate temperature, properties of ink solvent (including the volatility, viscosity and surface tension) and post annealing process are the three key parameters to affect the formation of thin films [65]. A moderate substrate temperature of 75°C resulted in improved surface wetting and reduction of non-uniformities. Tait *et al.* demonstrated the versatility of concurrently pumped spray coating for the optimization of multiple halide perovskite layers [66]. By adjusting the flow rate of PbCl_2_ and PbAc_2_ in combination with MAI, they obtained perovskite films with equivalent drying dynamics for each layer. As a result, perovskite films with full coverage and negligible pinholes were obtained, which led to a PCE of PSCs up to 15.7%. Heo *et al.* successfully obtained perovskite films with large grains (∼1.5 *μ*m) and low trap-state density by warming the substrate at 120°C and using a mixed solvent of DMF (154°C, 0.52 kPa) and GBL (204°C, 0.2 kPa) [29]. The reduced ink tension and contact angle of droplets on the substrate balanced the inward flux of the spray ink and outward flux of the evaporating solvent. As a result, a PCE of 15.5% for a sub-module (10 cm × 10 cm, active area = 40 cm^2^) was achieved.

The upscaling of PSCs induces several performance losses, in which the interfacial charge recombination and the interconnection loss of the multiple cells in a module are the most related reasons. Agresti *et al.* [30] spray-coated a graphene-doped TiO_2_ as ETL and functionalized molybdenum disulfide (F-MoS_2_) as an interfacial layer between perovskite and HTL to reduce the charge recombination reaction. The application of a spray-coated 2D interfacial layer led to PCEs of 13.4% and 15.3% for PSCs with active areas of 108 cm^2^ and 82 cm^2^, respectively. More recently, Rolston *et al.* reported scalable and fast open-air manufacturing of perovskite modules by combining the open-air spray and plasma deposition technique [28]. High performance modules (5.9 cm^2^) comprising 17 sub-cells with a stable power output of 15.2% were achieved, showing great potential toward large-scale in-line integration of perovskite solar technology. Shelf-life aging over 5 months indicated minimal degradation to unencapsulated modules stored in a dry environment, while continuous operational stability of 2 h in air for the unencapsulated module was reassuring. The authors suggested that additional interfaces introduced during laser scribing and direct contact between Ag and perovskite in the dead areas could result in a pathway for degradation.

## MODULE REPRESENTATION

For high performance perovskite modules, the primary requirement is to deposit a uniform perovskite layer with large grain size. By tuning the ink composition and printing technique, large-area uniform perovskite with a shining surface can be obtained by various solution-based techniques. As summarized in Table 1, most of the demonstrated PSCs with good performance are still in a small area defined by a mask, while the module efficiencies are relatively low, and far below the commercial request.

**Table 1.** PSC modules fabricated by various solution-based techniques.

Perovskite		PCE	*J*_SC_	*V*_OC_	FF	Active area	Operational stability		
components	Method	(%)	(mA/cm^2^)	(V)	(%)	(cm^2^)	(h)	Year	Ref

MAPbI_3_	Slot-die	21.8	24.79	1.10	80.3	0.1	0.17 (∼100%, -)	2020	[56]
	Blade coating	21.7	22.51	1.18	81.7	0.08	500 (>90%, -)	2019	[77]
	Inkjet printing	17.74	21.88	1.06	76.5	2.02	-	2018	[26]
AVAI-MAPbI_3_	Slot-die	12.1	21.4	0.96	59	70	Storage stability	2019	[78]
MAPbI_3−x_Cl_x_	Blade coating	20.2	22.7	1.10	81	0.08	Storage stability	2018	[79]
	Blade coating	15.38	19.84	1.17	66.4	10.08	300 (∼84%, -)	2020	[80]
	Slot-die	11.1	17.3	0.85	67.9	168.75	-	2020	[19]
MAPbI_3−x_Br_x_	Meniscus coating	15	-	-	-	25	-	2019	[81]
Unknown	Meniscus printing	11.7	-	-	-	703	-	2020	[82]
CsFAMAPbI_3−x_Br_x_	Inkjet printing	21.6	24.6	1.11	80	0.42	72 (∼100%, 25°C)	2019	[25]
CsFAMAPbI_3−x_Cl_x_	Slot-die	22.7	25.7	1.12	78.8	0.09	Storage stability	2020	[17]
		19.6	22.63	1.13	76.2	7.92			
FAMAPbI_3−x_Br_x_	Slot-die	15.6	19.5	-	-	36.1	1000 (∼91%, 60°C)	2020	[83]
	Meniscus printing	20.05	23.2	1.10	78.58	1	60 (∼90%, 25±2°C)	2017	[84]
	Blade coating	18.3	22.3	1.15	71.3	1	-	2018	[85]
RbCsFAMAPbI_3_	Blade coating	21.9	23.5	1.2	77.7	0.08	1000 (∼92%, -)	2020	[20]
CsFAMAPbI_3_	Blade coating	22	23.6	1.18	79	0.08	500 (∼96%, -)	2020	[86]
CsFAPbI_3_	Spray coating	15.2	20.6	1.06	71	5.9	12 (>99%, r.t.)	2020	[28]
CsFAPbI_3−x_Br_x_	Bar coating	17.01	22.3	1.08	70.5	18.66	-	2020	[40]

Apart from the uniformity and morphology of the perovskite layer over a large area, design and processing technologies also play an important role for development of a high performance solar module. Ho-Baillie and co-workers [67] reported a method of accurately modeling the effect of different front conductor structures on achievable solar cell efficiencies. For a small-area perovskite cell with a band gap of 1.48 eV, efficiency can reach 25% as simulated by the models. However, the maximum efficiency drops quickly as the cell width increases, limiting the efficiency to 19% when cell width is 2 cm (4 cm^2^ square cell), and <5% when cell width is 15.6 cm (1243 cm^2^ square cell) without metal grid or serial metal interconnection. The traditional approach to address this issue is to separate one large cell into many small sub-cells, all serially connected. This allows decrease of the serial resistance, thereby facilitating fabrication of high-performance mini-modules. Galagan *et al.* [68] simulated the current distribution in the modules with different widths of sub-cells to help optimize the modules. The results demonstrated that modules based on TCO substrates with sheet resistance of 10 Ω/sq have maximum performance with a sub-cell width of 5 mm. When using transparent PET/ITO substrate, the sub-cell width should decrease to 3 mm. Thus, there will be plenty of sub-cells in a large PSC module, resulting in an increasing number of interconnections (dead area), thus lowering the current density. The most promising technology to narrow the dead area and achieve a high geometrical fill factor (GFF) of the modules is laser ablation. A nano or picosecond laser ablation not only can minimize the dead area, but also enable selective ablation of the functional layer without damaging the layer underneath [69].

Besides research groups from university, there are several pioneer developers of PSC modules: Oxford PV, Microquanta Semiconductor, WonderSolar, Saule Tech., Panasonic, Toshiba, Solaronix, *etc.* [63]. Involvement of industry in technology development at an early stage ensures a fast transition from laboratory-scale fabrication toward industrial-scale manufacturing. However, for further development toward commercialization of perovskite PV, interest in and financial support for larger-scale projects are needed from both government agencies and industries at the present stage.

## PEROVSKITE INK ENGINEERING

For printing PSCs, three subjects are considered as the key issues to be addressed: ink engineering, printing strategies and patterning [70]. Of these three, inks have a significant role as ink preparation is the primary step to industrialize PSCs. The perovskite layer is the most important layer to achieve high-performance PSCs. To this end, it is vital to print high-quality films with good coverage, large grain sizes and preferential orientations [71,72]. The nucleation and crystalline growth mechanisms of perovskite on large-area substrates have been well-reviewed by many previous publications [33,48,73,74]. Therefore, in the following sections, we will mainly focus on development of perovskite inks and insights into ink chemistry for printing PSCs.

Generally, perovskite inks contain three main components, *i.e.* perovskite compositions, solvents and additives [75]. In terms of perovskite composition, a wide range of perovskite chemicals, such as pure-phase perovskite, mixed-anion and mixed-cation perovskites, 2D perovskites have been used for tuning the color/bandgap of the light-absorber, tailoring the film formation process, enhancing the stability, *etc.* [76]. Solvents are used to dissolve the components of the inks, adjust the viscosity and surface tension on substrates, and also influence perovskite nucleation and crystallization processes. Additives complete the required properties and functionality of the perovskite composition by modifying the ink properties to meet different requirements for printing methods and printed applications. At present, perovskite inks are under rapid development because of progress in synthesis of new materials and innovation in printing technologies. In the following sections, we will discuss ink engineering through these three aspects.

### Composition engineering

**Figure 3.**
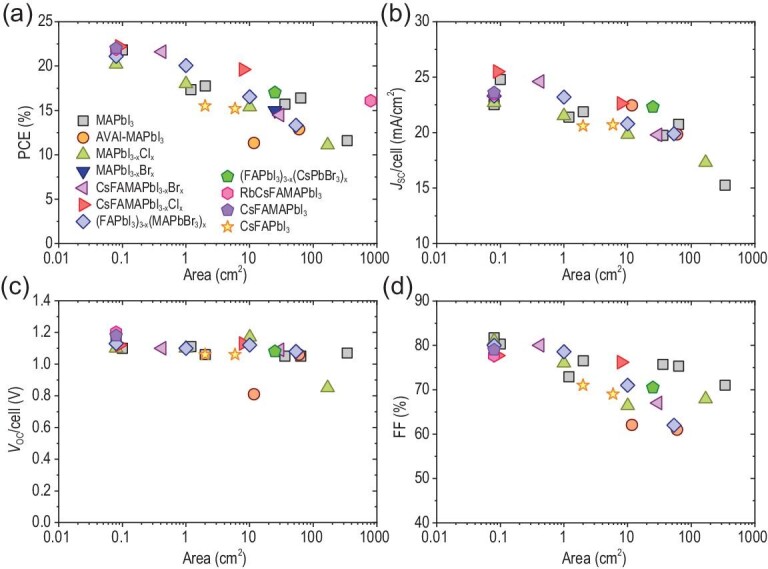
Photovoltaic parameters of the devices fabricated by scalable printing/coating method with respect to the active area for different perovskite compositions: (a) PCE, (b) *J*_SC_, (c) *V*_OC_ and (d) FF. The data are based on published works [9,17,19,20,23,25,26,28,40,54,56,77,79–81,84–93].

**Figure 4.**
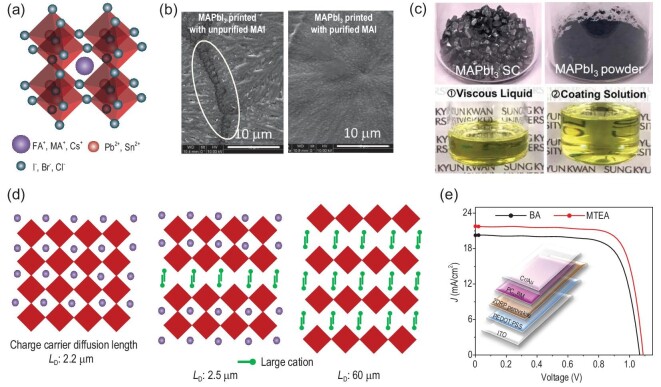
(a) Structural features of ABX_3_ perovskites. (b) SEM images of MAPbI_3_ films formed using unpurified and purified MAI. Adapted with permission from [94]. (c) Single-crystal or powder MAPbI_3_ was used as a starting precursor, which was first converted to viscous liquid in MA gas-filled closed vessel and then diluted with acetonitrile. Adapted with permission from [97]. (d) Schematic crystal structures and charge carrier diffusion length of CH_3_NH_3_PbI_3_, quasi-2D MA(PEA)_2_PbI_7_, and 2D (PEA)_2_PbI_4_ perovskite films. (e) The *J−V* curves of the PSCs based on (MTEA)_2_(MA)_4_Pb_5_I_16_ and (BA)_2_(MA)_4_Pb5I_16_ 2D perovskites. The inset is the device configuration. Adapted with permission from [118].

The composition of perovskite is directly associated to the film formation process, the bandgap and thus the device performance. In Fig. 3, we summarize the performance of perovskite cells and modules based on a range of mixed cations and halides perovskite, in which the perovskite layer was fabricated with a scalable printed/coated method rather than a spin coating process [9,17,19,20,23,25,26,28,40,54,56,77,79–81,84–93]. It is clearly indicated that a good PCE is achievable with most perovskite compositions *via* careful optimization of printing parameters and ink components. The composition of a perovskite can be tuned from pure, such as MAPbI_3_, FAPbI_3_ or CsPbI_3_, to mixed, such as (FA*_x_*MA*_y_*Cs*_z_*)Pb(I*_a_*Br*_b_*Cl*_c_*)_3_ (where *x *+ *y + z *= 1 and *a *+ *b *+ *c *= 1), as shown in Fig. 4a.

The purity of ink components is the first issue to be considered for film quality control. For example, the impurities (H_3_PO_2_, HI stabilizer) in synthesized MAI are of benefit to a spin-coated MAPbI_3_ device, whereas these are detrimental to blade-coated ones [94]. As shown in Fig. 4b, these impurities tend to accumulate at the edges of perovskite grains, leading to a non-continuous film with micrometer-wide gaps in between the grains. In contrast, with purified MAI, thick (∼1 *μ*m) and good coverage MAPbI_3_ films with large area (2.5 cm × 2.5 cm) were achieved, resulting in 15.1% efficiency of small-area PSCs. Chang *et al.* studied the impact of impurities in lead halide on perovskite film quality and device performance [95]. They found that traces of impurities, mainly metal salts in PbI_2_, not only provide recombination channels in devices, but also reduce the crystalline size of perovskite film (from 109 nm to 88 nm), which are both harmful to the device performance. To purify the perovskite compositions, Park *et al.* re-dissolved MAPbI_3_ single crystal by MA gas to prepare pure perovskite inks [96]. They demonstrated that films printed by the purified ink have better crystallinity and larger grain than those using ink made by precursor mixing, because of a variation of the chemical species (Fig. 4c) [97].

Another important issue that has been widely discussed is the perovskite precursor stoichiometry. It is often taken for granted that precursors should be combined in the final desired film stoichiometry, yet alterations to the stoichiometry may be more applicable to compensate for the loss of the volatile components, or to passivate interfaces or bulk defects [72]. Kim *et al.* reported that a small amount of excess PbI_2_ in perovskite could greatly improve the overall performance of PSCs [98]. Park *et al.* investigated the key features of the electrical and crystal properties of perovskite layers with and without excess PbI_2_, using techniques such as electron-beam-induced current (EBIC) measurements, grazing incidence wide-angle X-ray scattering (GIWAXS), and high resolution transmission electron microscopy (HR-TEM). They found that excess PbI_2_ influences the perovskite film through three main aspects: (1) it leads to reduced defects in the bulk film; (2) the PbI_2_-excess perovskite film features more ordered grains; (3) the presence of PbI_2_ within the grain boundaries of perovskite film can tailor energy band bending of the grain boundaries from downward to upward, reducing the charge carrier recombination [75,99]. Despite its potential to improve device performance, many studies found detrimental effects of excess PbI_2_ on film/device stability [100], solar cell parameters [101] and tetragonal–orthorhombic phase transition [102]. For example, Liu *et al.* [103] reported that excess PbI_2_ in MAPbI_3_ film caused an intrinsic instability of the film, leading to film decomposition under inert atmosphere and faster degradation on exposure to both illumination and humidity. On the other hand, Son *et al.* reported that 6 mol% MAI in the MAPbI_3_ precursor solution resulted in a perovskite film with high crystal quality, longer charge carrier lifetimes and high PL yields, which suppresses the non-radiative recombination and improves the hole and electron extraction at the grain boundaries [104]. Jacobsson *et al.* [105] demonstrated that in the PbI_2_-deficient samples the ion migration was obstructed, which decreased the *J−V* hysteresis and increased the photostability. Currently, most but not all the highest cell efficiencies were achieved based on excess lead or ammonium cation. Searching for new ways to yield the high crystal quality obtained under PbI_2_-deficient conditions while maintaining the favorable grain boundary characteristics obtained under PbI_2_-rich conditions, is a good strategy toward devices with high efficiency and stability.

MAPbI_3_ has been the most commonly used compound for printing perovskite films because of its relatively simple nucleation and crystal growth processes. Deng *et al.* certified a module efficiency of 16.4% (aperture area 63.7 cm^2^) with a MAPbI_3_ perovskite film, which is among the highest efficiencies of printed modules based on this perovskite [23]. The module retained 87% of its peak efficiency after 1000 h continuous operation at maximum power point (MPP), and no significant shading effect was observed. Although the PSCs based on MAPbI_3_ inks show high efficiencies, this compound tends to decompose at elevated temperature (>80°C) [106]. Other disadvantages, such as low reversible phase transition temperature (54–57°C), low moisture resistance, *etc.*, have urged researchers to pursue more stable perovskites [107]. FAPbI_3_ exhibits better thermal stability and a more ideal bandgap (≈1.1–1.4 eV) of 1.48 eV than MAPbI_3_ (1.55 eV) [107]. Chang *et al.* fabricated FAPbI_3_-based PSCs by dipping the printed PbI_2_ into the FAI solution, showing efficiencies >15% with an active area of 1.2 cm^2^ [108]. However, printing larger-area FAPbI_3_ has been challenging because of unstable *α*-phase at room temperature and also poor coverage on the substrate [109]. Mixing FAPbI_3_ with MAPbI_3_ can suppress phase transition and improve the film uniformity simultaneously [110]. By mixing these two perovskites at a ratio of 2 : 3, Deng *et al.* obtained PSCs with PCE more than 18% by a doctor-blade coating [111]. He *et al.* reported a large-grain and dense FA_0.85_MA_0.15_PbI_2.55_Br_0.45_ perovskite film with good crystallization and preferred orientation by a meniscus-assisted solution printing (MASP) strategy, achieving champion efficiency of 18.0% with an active area of 0.98 cm^2^ [84]. Thereafter, incorporation of different cations, such as Cs^+^, K^+^, Rb^+^ has also been reported, showing improved film quality and device performance in the printing processes. For example, Panasonic Corp. used a mixed perovskite ink (mixing MA^+^, FA^+^, Cs^+^ and Rb^+^) to achieve a PCE of 16.1% for a perovskite solar module (aperture area 802 cm^2^) by IJP technology [14]. Bu *et al.* reported a quadruple-cation perovskite absorber by mixing K^+^, Cs^+^, FA^+^ and MA^+^. They achieved a PCE of 15.2% for a flexible PSC module (with module size of 30 cm^2^) based on a slot-die-coated SnO_2_ ETL [54].

Recently, two-dimensional (2D) perovskites derived by isolating the corner-sharing lead halide octahedral sheets with long-chain or aromatic alkylammonium cations, have been demonstrated to be able to tune the perovskite crystallization process during the solvent drying process [112]. 2D perovskites have a generic structural formula of A^′^_2_A*_n_*_−1_BX_3_*_n_*_+1_, where B is the second cation and *n* represents the number of inorganic BX_6_ layers. They can self-assemble to form well-defined films on substrates, with good surface coverage and fine texture when the organic cation structure is carefully designed [113]. Furthermore, the bulky cations, lying between the inorganic layers, can also significantly enhance the moisture resistance of the 2D perovskite [114]. Such merits encourage the applications of 2D perovskites in printing processes. One important issue is that compared to 3D perovskites, the quantum well confinement effect in 2D perovskite leads to an increase in binding energy as a result of formation of excitons rather than free carriers [115]. Milot *et al.* reported that the effective charge-carrier diffusion length generally decreased with increasing 2-phenylethylammonium (PEA^+^) content, showing 2.2 *μ*m for MAPbI_3_ and 60 nm for (PEA)_2_PbI_4_ 2D perovskite [116]. With a trade-off between trap reduction, electronic confinement and layer orientation, the effective charge-carrier mobility of MA(PEA)_2_PbI_7_ quasi-2D perovskite can reach a maximum of 2.5 *μ*m (Fig. 4d). In addition, it was found that orientation of the 2D perovskite films strongly depends on the value of *n*, deposition procedures and substrate temperature. Tsai *et al.* found for larger-*n* (typically >3) Ruddlesden-Popper (RP) perovskites, the lead halide layers tend to lie flat on the substrate during film deposition, which allows carrier conductivity across the film in thickness direction [117]. Li *et al.* introduced 2D perovskite (EDBEPbI_4_) microcrystals into the MAPbI_3_ ink to template the crystal growth process [89]. High-quality perovskite films with larger grains and significantly reduced grain boundary defect were achieved. As a result, a device with EDBEPbI_4_ achieved a high PCE of 11.6% for large solar modules (342 cm^2^). Their result indicates that the molecular structure should be carefully considered upon printing 2D perovskite inks. For example, using 2-(methylthio)ethylamine hydrochloride as the large cation, Ren *et al.* obtained smooth, dense and pinhole-free 2DRP perovskite films with low trap state density resulting from the strengthened interlayer molecular interaction mediated by the sulfur–sulfur (S–S) interaction. As shown in Fig. 4e, a high-performance 2DRP-based PSC with a PCE of 18.06% and stability over 1000 h (85% initial performance remained) was achieved [118].

Anion substitution is also useful to optimize photoelectric properties and morphology of perovskite films, such as partial replacement of the I^−^ in APbI_3_ with Cl^−^ or Br^−^ [119]. Stranks *et al.* reported that the diffusion length of mixed halide perovskite MAPbI_3−x_Cl_x_ is greater than 1 *μ*m, which is an order of magnitude greater than that of MAPbI_3_ [120]. Tang *et al.* improved the reproducibility of doctor-blade-coated MA_0.6_FA_0.38_Cs_0.02_PbI_2.975_Br_0.__025_-based PSCs by introducing 5 mol% of Cl-compound into the perovskite ink [121]. The chlorine facilitates crystal growth and suppresses trap-state of the hybrid perovskite film, which is *via* a ‘mineral bridge’ mechanism [122]. Zhou *et al.* reported that fluoride can passivate the anion and cation vacancies in the perovskite films by strengthening the chemical bonds between lead and halides because of the strong electronegativity of fluoride [123]. Those works demonstrated that composition engineering is important to print/coat high-quality perovskite films with good stability.

### Solvent engineering for printing

Polar solvents, such as dimethylformamide (DMF), dimethyl sulfoxide (DMSO), γ-butyrolactone (GBL), N,N-dimethylacetamide (DMA), 2-methoxyethanol (2-ME), N-methyl-2-pyrrolidone (NMP) and acetonitrile (ACN) are common solvents used for perovskite inks [75]. By analyzing the boiling point, vapor pressure, coordination ability with lead halide, wetting and surface tension on substrate of the solvent, one can predict the printing processing windows such as the ink flow rate, substrate temperature and printing speed [124]. In the dissolution procedure, solvent molecules embed into interstices of the layered lead halide spontaneously, forming intermediate complexes of PbX_2_-solvent [125]. Those intermediate complexes determine the nucleation process and finally affect the morphology of perovskite films. In the printing/coating process, firstly a homogeneous wet film with a certain thickness is deposited. As the solvent keeps escaping, the wet film is supersaturated for nucleation and crystal growth of perovskites, and cations (MAI, FAI, *etc.*) embed into the lattice of PbX_2_ by pushing out the coordinated solvent molecules [126]. Therefore, the interaction between solvents and perovskite compositions is particularly important in the nucleation and crystallization processes during the perovskite film formation.

**Figure 5.**
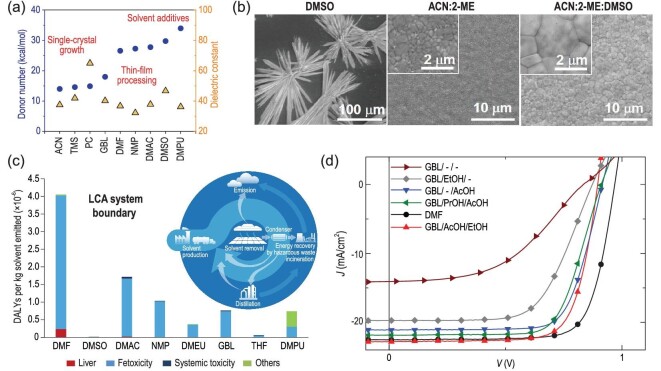
(a) Gutmann's donor number (*D*_N_) and dielectric constant of solvents [127] (including ACN, tetramethylsulfone (TMS), propylene carbonate (PC), GBL, DMF, N-methyl-2-pyrrolidone (NMP), dimethylacetamide (DMAC), DMSO, dimethylpropyleneurea (DMPU)) commonly used for PSCs. (b) Surface SEM images of perovskite films blade-coated with different solvent or solvent mixtures. Adapted with permission from [23]. (c) Human health characterization factors expressed in disability-adjusted life year (DALY) per kg of substance emitted for the scenario of emission to urban air. The inset is the life cycle analysis (LCA) system boundary schematic showing possible pathways for production of perovskite PVs. Adapted with permission from [128]. (d) *J−V* curves of blade-coated PSCs based on green solvent systems. Adapted with permission from [130].

The physical properties of the solvent, such as Mayer bond unsaturation, Hansen's solubility parameters, dielectric constant and Gutmann's donor number (*D*_N_) were used to define the interactions with component in perovskite ink. In general, solvents with dielectric constants >30 will have a good solubility for the perovskite precursors. However, Hamil *et al.* found that *D*_N_ was a stronger predictor of a solvent's solubility of perovskite precursor than the dielectric constant [127]. In addition, solvents with different *D*_N_ will alter the crystallization behavior of the perovskites. As shown in Fig. 5a, in solvents with low *D*_N_, halide dominates the coordination with Pb^2+^ and perovskites easily crystallize from the solvents, while solvents with high *D*_N_ compete for the coordination of I^−^ with Pb^2+^ to inhibit the crystallization of perovskites. Then higher *D*_N_ solvents can be employed as solvent additives to control crystallization of perovskite for fabrication of large-area dense perovskite films. Thus, the solvent mixture becomes an effective and general recipe to print/coat large-area perovskite films of good quality. Deng *et al.* reported a method of fast blade coating high-quality perovskite films at a speed of 99 mm/s *via* tailoring solvent coordination capability. As shown in Fig. 5b, by combining volatile non-coordinating solvents, *i.e.* ACN, 2-ME and low-volatile, coordinating solvent of DMSO, they obtained both fast drying and large MAPbI_3_ perovskite grains, delivering reproducible high-efficiency perovskite modules around 15–17% [23].

The toxicity of solvents, such as toxic DMF, skin penetrating DMSO or carcinogenic NMP is also an important point, especially for operators who have direct contact with volatile solvents in the printing process. Because of the inevitable use of abundant solvents, toxicity is an issue that must be resolved before mass-production of PSCs. Thus, searching for ‘green’ solvent systems could be a foundation to promotion of safe production. Recently, Vidal *et al.* analyzed the health and environmental impacts of eight solvents (shown in Fig. 5c) commonly used in perovskite deposition processes. By systematically considering the solvent production, use/removal, emissions and potential end-of-life treatments on an industrial scale, they claimed that DMSO is the most environmentally friendly and least deleterious to human health [128]. Noel *et al.* introduced methylamine to improve the solubility of MAPbI_3_ in ACN, full coverage and pinhole-free perovskite films as large as 125 cm^2^ and PCE of 19% for small-area PSCs were achieved [129]. As acetonitrile is currently used in a wide variety of industrial processes, this solvent system shows tremendous promise for safe fabrication of large-area perovskite films [97,129]. The reported green solvent systems are also compatible with the scalable solution deposition methods, such as D-bar coating. For example, Jeong *et al.* prepared a perovskite ink *via* a solid–liquid phase transition and dilution process, in which they dissolved MAPbI_3_ perovskite single crystal in methylamine gas and diluted with ACN. With a D-bar coating method, they achieved MAPbI_3_ film with highly oriented crystallization [97]. Gardner *et al.* successfully introduced a nonhazardous solvent/alcohol/acid system to the perovskite blade coating process, resulting in a device performance on a par with the standard hazardous inks (shown in Fig. 5d) [130]. Bu *et al.* used ethyl acetate in both perovskite anti-solvent engineering and spiro-OMeTAD HTL deposition process, which enabled a PCE up to 19.4% for lab-scaled small cells and 14.2% for a 5 cm × 5 cm module [131].

### Additives-engineering for printing

Incorporating additives into the precursor ink is an effective way to print high-quality perovskite films. Additives usually do not embed into the perovskite lattice structure, but they may affect film formation and have a large impact on the microstructure and/or defect population. Perovskite formation is a combination of nucleation and crystal growth processes. In the nucleation process, additives, such as hydrohalic acids, hypophosphorous acid and formic acid could enhance the film quality by promoting the dissolution of colloids in the perovskite ink [132]. As reported by Yan *et al.* [133], the perovskite ink is more like colloidal dispersions in a mother solution with a colloidal size up to the mesoscale, rather than real solutions. These colloid clusters provide nucleation sites for perovskite crystallization. A non-uniform distribution of large colloid clusters led to preferred crystallization in some sites but with a non-covered region between them, while smaller and more uniform colloidal particles resulted in better morphology with good coverage, crystallinity and texture. The crystal growth process can also be manipulated by forming intermediates rather than perovskite directly. For example, pseudohalide thiocyanate ions (SCN^−^), which have a similar ionic radius to I^−^ and the capability of forming strong interactions with Pb^2+^ because of the lone pair electrons from S and N atoms, have been widely used in 3D perovskites to modulate the crystallization kinetics.

**Figure 6.**
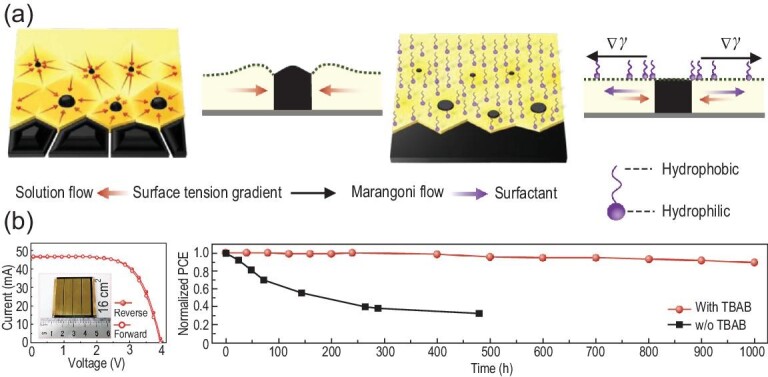
(a) Solution flow dynamics and its suppression by surfactants. Schematic illustration for the directional microscale solution flow towards perovskite island during ink drying and the suppressed solution flow dynamics in the presence of surfactant. *Δγ*, surface tension gradient. Adapted with permission from [44]. (b) TBAB additive modulated large area perovskite film coating for high performance modules: *J−V* curves of the best performing module with the size of 16 cm^2^ measured from reverse and forward scan under AM 1.5G. The active area of the module was estimated to be 8.0 cm^2^. Left: the inset is a photo of the module; right: the long-term behavior of the modules based on the perovskite films tailored by TBAB. Adapted with permission from [136].

The additives can be incorporated into perovskite films in two ways: (1) direct addition into the precursor ink; (2) introduction through post treatment. The first not only affects the nucleation and crystal growth process but also passivates the defects, whereas the second primarily passivates the defects [134]. As shown in Fig. 6a, Deng *et al.* reported an innovative route to modulate the crystallization of perovskite films on large-area substrate during a blade coating process. They added L-*α*-phosphatidylcholine to the perovskite ink as a surfactant to suppress the fluid and particle flow during the drying of the wet film, resulting in uniform perovskite films and a module (57.2 cm^2^, aperture area) with stable power output efficiency of 14.6% [44]. Rong *et al.* reported that additives, such as ammonium chloride (NH_4_Cl), and moisture form an intermediate of MAX·NH_4_PbX_3_(H_2_O)_2_ (X = I or Cl) with perovskite components during the film formation process, in which the intermediate firstly retards the crystallization of perovskite and then induces the transition of intermediate to perovskite phase [135]. As a result, high-quality perovskite MAPbI_3_ crystals with preferential orientation were obtained in the screen-printed triple-layer scaffold of TiO_2_/ZrO_2_/carbon, leading to an efficiency of 15.6% and a lifetime over 130 days for PSCs.

For this purpose, many chemicals (see Table 2), such as Lewis acids/bases, ammonium or inorganic salts, were introduced into perovskite ink to produce high-quality perovskite films and high-performance PSCs [72]. It was reported that the additives modulate the morphology and electronic properties of the final perovskite films by changing the solution chemistry and participating in nucleation and crystal growth processes through hydrogen bonds, coordinate covalent bonds or forming an intermediate complex with perovskite components in the ink [132,134]. For example, Xu *et al.* incorporated a Br-containing tetrabutylammonium salt (TBAB) into the perovskite precursors, which demonstrated superior advantages in both crystallization and large-area uniformity control during the blade coating process of perovskite films [136]. As shown in Fig. 6b, the modified module with four sub-cells showed efficiency of 15.8%, and a remarkable ambient stability towards moisture was achieved, retaining over 89% of its initial efficiency after 1000 h with a humidity of about 45% at room temperature (25°C).

**Table 2.** Additives involved in PSCs fabricated with scalable techniques.

Additive components	Method	Perovskite components	PCE (%)	*J*_SC_ (mA/cm^2^)	*V*_OC_ (V)	FF (%)	Active area (cm^2^)	Operational stability (h)	Year	Ref

L-*α*-phosphatidylcholine	Blade coating	MAPbI_3_	15	19.5	1.07	72.1	33	Storage stability	2018	[44]
F4TCNQ	Blade coating	MAPbI_3−x_Cl_x_	20.2	22.7	1.1	81	0.08	Storage stability	2018	[79]
PEO	Slot-die	MA_0.6_FA_0.38_Cs_0.02_PbI_2.975_Br_0.025_	11.62	16.8	0.92	76	0.1	-	2019	[52]
BAA	Blade coating	MAPbI_3_	20.0	22.0	1.14	80	1.1	500 (>90%, -)	2019	[77]
TBAB	Blade coating	MAPbI_3−x_Cl_x_	19.34	23.24	1.07	78	0.12	400 (∼100%, -)	2019	[136]
			15.79	5.82	3.98	68	8	Storage stability		
Zinc porphyrin	Blade coating	MAPbI_3_	18.3	22.64	1.09	74	1.96	Storage stability	2019	[21]
GAI/MAAc	D-bar coating	MAPbI_3_	19.44	21.3	1.189	77.8	0.125	-	2019	[137]
			13.85	-	-	-	16			
S-benzyl-L-cysteine	Blade coating	MAPbI_3−x_Cl_x_	15.38	4.96	4.67	66.4	10.08	300 (∼84%, -)	2020	[80]
4-tert-butylpyridine	Blade coating	FA_0.85_MA_0.15_PbI_2.55_Br_0.45_	16.54	3.47	6.71	71	10	Storage stability	2020	[93]
			13.32	1.8	11.83	62	53.6			
HMPA	D-bar coating	(FAPbI_3_)_0.875_(CsPbBr_3_)_0.125_	18.07	22.61	1.07	74	0.125	-	2020	[40]
			17.01	2.23	10.81	70.5	18.66			
5-AVAI	Slot-die	MAPbI_3_	12.87	2.48	8.5	61	60.08	-	2020	[90]

Lewis acids/bases are defined as accepting and donating of electron pairs, respectively. For example, Lewis-basic additives were used to form adducts with lead halides to increase the solubility of the latter, as well as modify the annealing process of printed films [138]. The weak chemical interaction in the adduct slows down the crystallization speed of perovskites, thus widens the operating window for the printing process. Lewis acid and base molecules such as triphenylphosphine oxide [139], thio-semicarbazide [140] and 3-(5-mercapto-1H-tetrazol-1-yl)benzenaminium iodide [141] were also used to optimize the morphology of perovskite and therefore improve the device efficiency, because of their multifunction including recombination reduction, phase segregation suppression and moisture resistance. Li *et al.* introduced monoammonium zinc porphyrin (ZnP) into the ink to form an ionic bond with perovskite grains, which led to a champion PCE of 18.3% (1.96 cm^2^) *via* blade coating [21].

Similar to Lewis acid and base molecules, polymers featured with function groups have been used as additives in the perovskite printing processes. Generally, the polymer additives are added into the precursor ink and located at the grain boundaries after the annealing, thus they stabilize perovskite crystals and resist the water erosion. Chen *et al.* reported that the incorporated poly(bithiophene imide) (PBTI) can weakly bond with perovskite components (Pb^2+^ or I^−^) in the perovskite film, thus suppressing the ion migration and grain boundary degradation of perovskite [142]. Kim *et al.* used PEO as an additive in the precursor ink to improve the tolerance of perovskite in high humidity conditions for ambient deposition. They achieved 11% PCE of flexible PSCs with this additive-containing perovskite ink through a R2R slot-die coating process [52].

## R2R SCALING-UP

Toward commercialization, the R2R process has shown promise because of its fast mass production ability. The advantages of R2R printing in terms of volume, robustness and reproducibility have been well demonstrated in organic solar cells [143]. Similarly, R2R printing can be employed for fabricating PSCs. In contrast to print processing on a rigid substrate, R2R manufacturing intrinsically requires substrates and products to be bendable at least to some degree, which increases the difficulty in preparing high-efficiency PSCs. Flexible PSCs possess their unique merits such as light weight and flexibility, expanding the applicability of PSCs into a variety of mobile electronic devices, building- and vehicle-integrated solar cells. In this section, we provide a brief overview about current development on flexible PSCs that can be potentially printed *via* the R2R process.

A flexible substrate is a prerequisite for R2R printing. There are three main types of substrates that have been used for flexible PSCs: polymer films, metal foils and ultra-thin flexible glasses. Polyethylene terephthalate (PET) and polyethylene naphthalate (PEN) are the two of the commonly used polymer substrates, because of their high transparency and flexibility. To date, most of the high-efficiency flexible PSCs are achieved on PET or PEN substrates. In 2019, Zhu *et al.* demonstrated flexible PSCs on PET with a PCE of 18.5% [144], and the efficiency was improved to 21.1% by Yang *et al.* based on PEN substrate [145]. Colorless polyimide (CPI), having a much higher thermal tolerance than that of PET and PEN, has been used as another alternative substrate for flexible PSCs [146]. Thin metal foils (including stainless steel and titanium) are also commonly used as favorable substrates for flexible solar cells [147], which can provide strong mechanical property and good tolerance to high temperature. Han *et al.* oxidized a layer of TiO_2_ on titanium foil and used this as a substrate for flexible PSCs [148]. The device achieved a PCE of 14.9% and exhibited high resilience to bending test with no efficiency loss after 1000 bends. The main disadvantage of the metal foils is their opaque nature, requiring another transparent electrode in the rear of the cell. Ultra-thin flexible glass combined the advantages of both the polymer and metal foils, making it an ideal candidate for a flexible PSC substrate [149]. Dou *et al.* [150] demonstrated flexible PSCs with a PCE of 18.1% on ultra-thin flexible glass with conducting indium zinc oxide (IZO).

**Figure 7.**
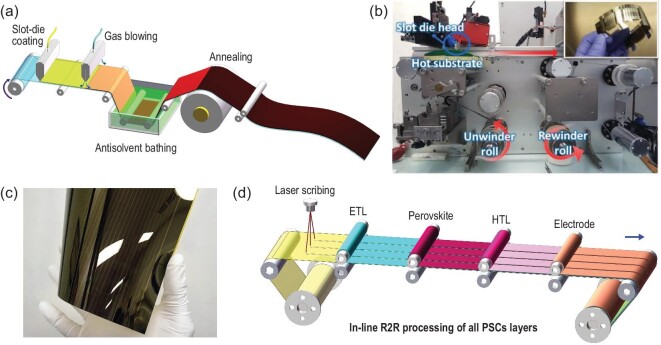
(a) Diagram showing R2R-compatible treatments (including gas-blowing, antisolvent bathing and annealing) for perovskite layer. (b) Photos of the slot-die-coater equipped a R2R machine, and the flexible PSCs fabricated by this machine. Adapted with permission from [52]. (c) R2R printed perovskite solar module demonstrated by Solliance. Adapted with permission from [155]. (d) Schematic illustration of in-line R2R processing of all PSC layers.

Despite high PCEs being achieved in PSCs with various flexible substrates, the production methods of these high-efficiency devices are not fully compatible with R2R processing. Schmidt *et al.* demonstrated one of the first flexible PSCs fully printed by the R2R method. The perovskite layer was deposited by slot-die coating and the as-prepared flexible PSC exhibited a low PCE of only 4.9% [151]. The gas-blow and anti-solvent quenching methods, which have been widely employed as post treatment for spin-coated perovskite film, have been gradually developed to combine with the R2R printing process (see Fig. 7a). Zuo *et al.* demonstrated a R2R compatible blowing-assisted drop-casting (BADC) method to prepare MAPbI_3_ films on PET substrate [51]. The coating unit comprised a slot-die head and a N_2_ blowing head. Two hot plates were used to dry and anneal the film. The final quality of the film was controlled with the complex coordination of a range of parameters including web speed, solution feeding rate, hot plate temperature, and distance between coating head and N_2_ blower, as well as the N_2_ flow rate. After optimization, they achieved a high PCE of 11.16% on flexible PSCs. After a short while, Kim *et al.* from the same group demonstrated that, by simply heating up the coating bed to 130°C during printing, the crystallinity of the R2R-printed perovskite layers could be improved. As shown in Fig. 7b, a curved heater was placed under the slot-die head during hot slot-die coating. The substrate continuously moved, allowing preheating for 10 s at 130°C before it reached the slot-die head. The champion device showed a promising PCE of 11.7%, which was a record PCE for R2R-processed PSCs at the time [52]. Galagan *et al.* also presented a dedicated optimization of the R2R drying and annealing conditions and declared that their PCE could be higher than 14.5% [152]. Very recently, Kim *et al.* successfully combined the antisolvent bathing with R2R process, and demonstrated manufacturing of a 100-meter-long roll. The fully R2R-printed flexible PSCs, except for top electrode, achieved a high PCE exceeding 16% [41]. Flexible devices with only gravure-printed perovskite layer possessed a high PCE up 19.1%, which is comparable to the spin-coated version. Apart from the academic institutions, several companies including Energy Materials Corporation (EMC), Coring Inc., Kodak and Solliance have also shown great interest in R2R-printed PSCs [153–155]. In 2019, EMC teamed up with Eastman Kodak to lease its high-speed R2R printers, the same printers used to make photographic film, to fabricate PSCs. The printer can coat 1000 feet of film per minute, and EMC hopes to coat perovskite at 100 feet per minute, a speed that could produce enough solar panels to generate 4 GW of electricity per year. Besides, with a unique R2R printing line, which is capable of multi-layer slot-die printing of solution-based layers, the research team at Solliance succeeded in producing flexible PSC modules with PCE of 12.6% (Fig. 7c) [155].

The enduring research work of academia and industry into PSCs means that printing perovskite thin film *via* the R2R process is no longer the main restrictive factor for high-performance flexible PSCs. Therefore, to speed up the development of PSCs to high-throughput technology, the fabrication of other functional layers should be adapted to R2R. At the current stage, the printable electrode is the bottleneck of developing fully R2R-printed PSCs. Although silver nanowires [156], carbon nanotubes [157], graphene [158] and organic materials [159] have shown potential ability to perform as the top electrode for flexible PSCs, most of these were used to replace the conducting oxide rather than the metal electrode. Apart from the electrode, more effective processing techniques also need to be developed along with the new environmental and processing stable materials (including ETL and HTL materials) that can sustain ambient processing conditions. In an ideal R2R technique of in-line processing, all of the PSCs layers are processed sequentially during the same run (Fig. 7d). This implies that the full integration of the current initiatives and subsequent developments needs to be performed at higher scale and faster pace if this technology has to be commercialized. In the long term, the parameters such as solvent viscosity, boiling point, toxicity and environmental compatibility of all the materials must be considered for better device performance and production efficiency.

## STABILITY

**Figure 8.**
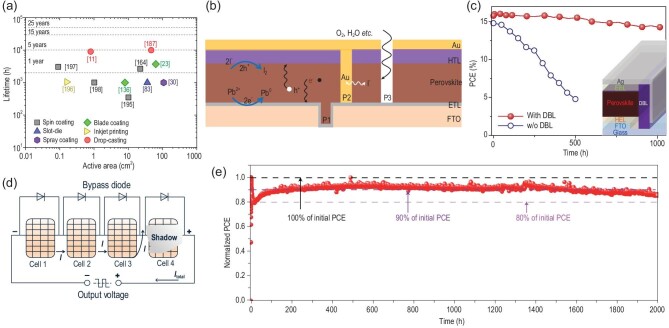
(a) Distribution of the lifetimes of PSCs and modules in terms of the device area fabricated with different printing or coating techniques. The data are based on published works [11,23,30,83,136,164,187,195–198], and the average sunshine duration time per year is set to 2000 h. (b) Stability analysis of the PSC module: the direct contact between perovskite and metal electrode in P2 patterning lines, extra channels for ions diffusion and water erosion induced by P3, and the light-induced cathode and anode reaction. (c) The performance of encapsulated perovskite modules under 85°C with 85% RH for 1000 h w/o DBL. Adapted with permission from [83]. (d) Schematic representation of a series of connected perovskite module under partial shade and bypass diode. (e) A holistic approach to interface stabilization for perovskite modules. Adapted with permission from [164].

Stability is crucial for ensuring the commercial success of perovskite PV technology. As reported by Domanski  *et al.* [160], the instability of PSCs can be distinguished by two aspects, *i.e.* extrinsic and intrinsic parameters. The former are harmful environment factors, such as exposure to high intensity visible [161] and ultraviolet (UV) light [162] or contamination from ambient environment (oxygen, humidity), which ideally can be blocked by encapsulation. The latter, *i.e.* high temperature and electrical bias, is crucial as certain materials might be excluded from being employed in long-term stable PSCs [163]. As seen in Fig. 8a, the T_90_ (the time over which the device efficiency reduces to 90% of its initial value) of PSCs based on a screen-printed perovskite reaches almost 5 years (2000 h sunshine duration per year), while that for perovskite mini-modules under continuous operation is less than 1570 h [164]. The difference in lifetimes between perovskite cells and modules is large, with both clearly lagging behind the silica PV products. The reason is that the degradation mechanism of perovskite modules is affected by more parameters whose impact on device stability is still elusive because of their particularities, such as the presence of mobile charged species [165], hysteresis in *J*−*V* characteristics [166], different recovery processes after stress removal, *etc.* [167,168]. More importantly, most previous stability assessment on PSCs is based on small-sized cells (active area ∼0.1 cm^2^), which are significantly different from modules [169]. First, the non-uniformity issues of the perovskite and charge transporting layers will be more pronounced on the enlarged substrate [69]. Second, perovskite modules are composited by multiple series connected sub-cells and the P1, P2 and P3 interconnection structures. The P2 and P3 patterning lines also increase the possibility of degradation (see Fig. 8b), such as the direct contact between perovskite and metal electrode, and extra channels for ion diffusion [31]. In addition, in a partially shaded module, the shaded sub-cells are subjected to a high reverse bias, which could lead to local hot spots and inverted-bias junction damage [170]. These same effects might also couple to intrinsic and extrinsic ionic conduction phenomena to change the module performance over time [163]. Therefore, degradation of PSCs at the module level is more complicated than single cell. For example, Luther *et al.* studied the degradation behavior of perovskite cells and modules under constant operation in a nitrogen atmosphere. The mini-modules containing four sub-cells retained ∼92% of the initial PCE after 100 h of constant operation, while the similar small-area single-cell conducted in the same environment retained 98% after 1500 h of testing [171,172]. In the following sections, the typical factors that affect the stability of perovskite cells and modules will be discussed.

### Light illumination

Light soaking promotes ion and defect migration in PSCs [173,174] as well as phase segregation [175,176] in the mixed perovskite layer, which decreases efficiency. Additionally, light can induce or accelerate harmful chemical reactions at the interfaces, which lead to detrimental changes in organic charge extraction layers, material intermixing at the interfaces and ion exchange between adjacent layers [177,178]. Sekimoto *et al.* [177] found that after light illumination, accumulation of iodine and metallic lead in the vicinity of the perovskite/HTL and ETL/perovskite interfaces, respectively (see Fig. 8b). They proposed four countermeasures to completely solve the light stability issue of PSCs: (1) suppressing the cathode reaction at the ETL/perovskite interface using a highly halide-resistant ETL or interfacial layer; (2) suppressing halide migration within the perovskite layer; (3) suppressing the anode reaction at the perovskite/HTL interface using a highly halide-resistant HTL or interfacial layer and (4) suppressing the interface charge accumulation by improving the hole/electron extraction capability. Recently, Wang *et al.* simultaneously suppressed the anode and cathode reactions by incorporating europium (Eu) ion pairs within the perovskite layer [179]. The Eu^3+^/Eu^2+^ pair acts as a redox shuttle that selectively oxidizes Pb^0^ to Pb^2+^ and reduces I^0^ to I^−^ in a cyclical transition, resulting in 92% retention of the peak PCE under one sun continuous illumination after 1500 h.

### Oxygen and humidity

Most PV materials suffer from corrosion in the presence of moisture or oxidation in the presence of oxygen, such as active materials and metal electrodes. Ideally, oxygen and humidity can be fully blocked by a glass-glass encapsulation. However, the solar cells will likely be exposed to ambient environment as they need to be transported through air in a factory before they are encapsulated. Therefore, understanding the degradation mechanism and searching perovskite materials with high intrinsic stability to moisture and oxygen is of great interest. Bryant *et al.* reported that the MAPbI_3_ perovskite degrades rapidly in the presence of a combination of light and oxygen [180]. Under illumination, molecular oxygen can react with the photogenerated electrons within the perovskite layer to form superoxide O_2_^−^, which deprotonates MA^+^ and eventually decomposes the perovskite into PbI_2_ and methylamine gas. Aristidou *et al.* [181] suggested that perovskite films composed of large crystallites with low defects show low yields of superoxide and high stability.

The critical role that moisture plays in the degradation of perovskites was identified early on in their development [182]. Leguy *et al.* proposed that moisture induces reversible and irreversible degradation of perovskite films as follows [183]:

(5) \begin{equation*}\begin{array}{@{}*{1}{c}@{}}{4{\rm{ C}}{{\rm{H}}_{\rm{3}}}{\rm{N}}{{\rm{H}}_3}{\rm{Pb}}{{\rm{I}}_3} + {\rm{ }}4{{\rm{H}}_2}{\rm{O}}}\\ {\leftrightarrow 4\left[ {{\rm{C}}{{\rm{H}}_3}{\rm{N}}{{\rm{H}}_3}{\rm{Pb}}{{\rm{I}}_3} \cdot {{\rm{H}}_2}{\rm{O}}} \right]}\\ {\leftrightarrow {{\left( {{\rm{C}}{{\rm{H}}_3}{\rm{N}}{{\rm{H}}_3}} \right)}_4}{\rm{Pb}}{{\rm{I}}_6} \cdot 2{{\rm{H}}_2}{\rm{O }} + {\rm{ }}3{\rm{Pb}}{{\rm{I}}_2} + {\rm{ }}2{{\rm{H}}_2}{\rm{O}}}. \end{array}\end{equation*}

On the other hand, Huang *et al.* reported that a certain amount of H_2_O can facilitate nucleation and crystallization processes of the perovskites, resulting in high-quality perovskite film and enhanced PSC performance [182]. The perovskite materials, including both films and single crystals are irreversibly destroyed by H_2_O after exposure to a high level of H_2_O. Therefore, humidity-resistant fabrication of high-performance PSC devices and modules should be favored. Xu *et al.* reported remarkable air stability of perovskite modules toward moisture by introducing alkylammonium cations of tetrabutylammonium into perovskite films. The perovskite module containing four sub-cells retained over 89% of its initial efficiency after 1000 h at a humidity of ∼45%, while the control module had a rapid decline to <30% after aging for 480 h.

### High temperature

As pointed out by Boyd *et al.* [184], within the fabrication and operation processes, a perovskite module might degrade by high temperature in three scenarios: (1) perovskite films and/or charge transporting layer annealing at high temperature >100°C, (2) high temperature encapsulation >140°C, (3) device operating temperature reaching up to 85°C. Additionally, evaluation of the stability of the PSCs at high temperature accelerates the electro-chemical reactions and degradation processes, which is helpful to shorten the testing time [185]. It is worth noting that most thermos-gravimetric analyses (TGA) of perovskite powder, *i.e.* MAPbI_3_ show no mass loss at annealing temperatures (<140°C) or operational temperatures (<65°C), while PbI_2_ could form within 24 h when heating MAPbI_3_ film in ambient at 85°C [106]. Results of *in situ* synchrotron grazing incidence X-ray diffraction indicated that decomposition of perovskite film at these moderate temperatures is more related to defective surfaces and interfaces, which have much lower energetic barriers to decomposition [186]. As discussed above, substitution of the volatile MA^+^ by FA^+^, Cs^+^, Rb^+^ or large cations in the perovskite films can improve the resistance to decomposition at operational temperatures. For example, impressive stability results have been reported by Grancini *et al.* for an encapsulated mini-module based on the TiO_2_/ZrO_2_/perovskite/carbon architecture, which incorporated a low dimensional perovskite as photo-active layer [187]. On the other hand, passivation and encapsulation of the surface of the perovskite films form an effective strategy to improve their thermal stability [188]. Han *et al.* introduced tri-s-triazine-based graphitic carbon nitride (g-C3N4) as low-dimensional diffusion barriers (DBLs) on the perovskite layer to block the ion diffusion channels, which enabled a PSC module to maintain 95% of the initial PCE after 1000 h under heating aging at 85°C (see Fig. 8c) [83].

### Electrical bias

Electrical bias-induced degradation is crucial as PV cells always operate under bias, typically near their maximum power points (MPP). Partial shade (shaded by clouds, water, dirt, *etc.*) sub-cells may experience reverse bias to match the current flow through connected illuminated cells [189]. The large current density can cause excessive local heating, damage to the cell and/or the encapsulant, hot spots from local shunts, *etc*. A first detailed study on the topic of reverse bias stability in PSCs was reported recently by Bowring *et al.* [190]. The authors demonstrated that PSCs with both *p-i-n* and *n-i-p* configuration degraded under moderate reverse voltages (less than −1 V) with hot spots appearing in just a few minutes because of proposed electrochemical reaction between the perovskite photoactive layer and charge transporting layers. Khenkin *et al.* [163] reported that extrinsic and intrinsic migration of mobile ionic species under electrical bias is one of the main reasons for PSC instability, which is also known to instigate degradation of CdTe and CIGS-based photovoltaics. Razera *et al.* [191] studied the degradation behavior of *p-i-n* PSCs based on FA_0.83_Cs_0.17_PbI_2.49_Br_0.51_ perovskite. They found that three main degradation processes occur during the reverse bias testing: (i) voltage-driven shunt formation, forming highly conductive shunts in regions covered by the metal electrode; (ii) ion migration-induced series resistance increase, electrochemical reaction between the perovskite absorber and adjacent layers, causing S-shape in the *J−V* curve and lowering PCE [190]; (iii) phase segregation under heavy reverse bias, degrading the perovskite absorber into iodide- and bromide-rich sub-layers. The voltage-driven shunting and phase segregation accompanied by microstructural changes are irreversible, while the halide movement is, to some extent, reversible. They proposed that other than improve the cells resilient against reverse bias, bypass diodes are also recommended to limit the reverse voltage to safe values (see Fig. 8d). Thus, a careful bypass strategy is needed to minimize the number of necessary bypass diodes and, consequently, module cost [192].

Market dominating crystalline silicon PV offers a linear degradation rate of ∼0.50% power reduction per year giving a power output of 80–85% of initial value after 25 years. Therefore, PV market and manufacturers suggest that any new PV technologies should fulfill similar degradation rates to ensure a competitive levelized cost of electricity (LCOE) [193]. The IEC61215:2016 is a generally used qualification standard for silicon PV and includes requirements to undergo mechanical, thermal and environmental tests with a specific maximum level of degradation allowed, which is now widely used to evaluate the lifetime of emerging solar cells. There has been substantial progress in improving the stability of PSCs during the past few years because of better understanding of the degradation mechanisms, innovations of new materials, fabrication techniques, encapsulations, *etc*. Recently, Ho-Baillie and co-workers reported that through a low-cost polymer/glass stack encapsulation, the typical *n-i-p* PSCs passed the Damp-Heat and Humidity-Freeze tests, which are supposed to be the most critical parts of the IEC61215:2016 [194]. Han *et al.* reported that their fully printed PSCs with triple-layer scaffold of TiO_2_/ZrO_2_/carbon successfully passed the IEC61215:2016 [11]. Liu *et al.* [164] reported a perovskite solar module (area 22.4 cm^2^) retained ∼86% of the initial performance after continuous operation for 2000 h under AM1.5G light illumination (see Fig. 8e). These promising results are encouraging, but more studies and progress on the perovskite modules rather than a cell level are expected, which will drive the way for the perovskite photovoltaic manufacture with long-term stability in diverse aspects.

## PERSPECTIVE AND CHALLENGES

Going forward, the solar industry has clear cost-reduction and efficiency improvement roadmaps, which should see solar energy costs halving by 2030. In the last few years, impressive progress has been achieved in the quality of perovskite films as well as the efficiency of large-area PSCs. However, the performance of the perovskite modules is still notably lagging behind the small-area cells. On one hand, deposition of high-quality perovskite films in large scale is of critical importance to bridge the efficiency gap between small-area devices and large-area solar modules. On the other hand, challenges in scaling-up PSCs involve developing scalable printing techniques for all device layer stacks, including the perovskite layer, ETL, HTL and electrodes, as well as reliable module design (laser-scribing process, interconnection, width of sub-cells, *etc.*). PSCs have been proven to have the capability of high-volume production achieved *via* various printing techniques, which have been developed over years in the field of organic electronics, such as OPV, OLED and OFET. There are many similarities in the processes in terms of ink preparation and wet film printing/coating processing. In contrast to printing of other electronic or optoelectronic materials, such as metal nanoparticles, small organic molecules, or polymers, in which formation of the film depends essentially on solvent evaporation, nucleation and crystal growth in the printing process is of utmost importance for perovskite thin films. During the printing process of perovskite, wet thin films are fabricated from inks that are made of precursor chemicals, such as lead halide and ammonium halide salt, and subsequent to crystallize to perovskite structure. Therefore, apart from the general considerations of printing techniques, consideration of the nucleation and crystal growth processes during perovskite printing are further required.

The PSCs must also fulfill a rather high order of being stable, sustainable and able to compete with existing technologies on price. Respectable progress has been made over recent years to enhance the stability of PSCs by demonstrating long-term stability over 3000 h under illumination [195–198]. Further advances in terms of stability, particularly the demonstration of solar module stability, are needed to lift the technology to a level where it is ready to compete with, or be a companion to the current silicon solar cells.
